# Extraction Optimization of Phenolic Compounds from *Triadica sebifera* Leaves: Identification, Characterization and Antioxidant Activity

**DOI:** 10.3390/molecules29143266

**Published:** 2024-07-10

**Authors:** Shao-Jun Fan, Xin-Yue Zhang, Yu Cheng, Yu-Xian Qiu, Yun-Yi Hu, Ting Yu, Wen-Zhang Qian, Dan-Ju Zhang, Shun Gao

**Affiliations:** 1 Department of Forestry, Faculty of Forestry, Sichuan Agricultural University, Chengdu 611130, Chinahuyunyi@stu.sicau.edu.cn (Y.-Y.H.);; 2 School of Life Science and Engineering, Southwest Jiaotong University, Chengdu 611756, China; yuting212916@my.swjtu.edu.cn; 3 National Forestry and Grassland Administration Key Laboratory of Forest Resources Conservation and Ecological Safety on the Upper Reaches of the Yangtze River, Sichuan Agricultural University, Chengdu 611130, China

**Keywords:** extraction optimization, response surface methodology, phenolic compounds, leaf waste, HPLC, antioxidant activity

## Abstract

*Triadica sebifera* (*T. sebifera*) has attracted much attention because of the high oil content in its seeds, but there are few systematic studies on the phenolic compounds of *T. sebifera* leaves (TSP). In this study, the extraction process of TSP was optimized by response surface methodology. The phenolic components of these extracts were analyzed by high-performance liquid chromatography (HPLC). Moreover, the effects of hot air drying (HD), vacuum drying (VD) and freeze drying (FD) on the antioxidant activity and characterization of *T. sebifera* leaf extract (TSLE) were evaluated. Under the conditions of ethanol concentration 39.8%, liquid–solid ratio (LSR) 52.1, extraction time 20.2 min and extraction temperature 50.6 °C, the maximum TSP yield was 111.46 mg GAE/g dw. The quantitative analysis and correlation analysis of eight compounds in TSP showed that the type and content of phenolic compounds had significant correlations with antioxidant activity, indicating that tannic acid, isoquercitrin and ellagic acid were the main components of antioxidant activities. In addition, through DPPH and ABTS determination, VD-TSLE and FD-TSLE showed strong scavenging ability, with IC50 values of 138.2 μg/mL and 135.5 μg/mL and 73.5 μg/mL and 74.3 μg/mL, respectively. Fourier transform infrared spectroscopy (FTIR) and scanning electron microscopy (SEM) infrared spectroscopy revealed small differences in the extracts of the three drying methods. This study lays a foundation for the effective extraction process and drying methods of phenolic antioxidants from *T. sebifera* leaves, and is of great significance for the utilization of *T. sebifera* leaves.

## 1. Introduction

*Triadica sebifera* (*Linnaeus*) *Small* (*T. sebifera*), a deciduous plant of the Euphorbiaceae family native to China, is widely distributed in several tropical and subtropical regions of the world, including Japan, India, Vietnam, Europe, Africa and the Americas, etc. [1,2]. In China, it is mainly distributed in the provinces and districts south of the Yellow River. It is one of the four major woody oilseed crops in China with good adaptability and easy cultivation [3]. *T. sebifera* seeds have a high oil content, so they are widely used in the manufacture of candles, soaps, varnishes and paints, and are regarded as a high-quality raw material for biodiesel and biomass production [4,5,6]. In actual production, people only focus on the utilization of *T. sebifera* seeds, and treat the leaves as waste, which results in a serious waste of resources.

Studies have shown that the extracts of *T. sebifera* leaves (TSLs) exhibit a variety of biological activities, such as antibacterial, anti-inflammatory, antihypertensive, analgesic, etc., and have been used in traditional medicine to treat eczema, shingles, and edema, etc. [7,8]. *T. sebifera* leaves are rich in natural bioactive compounds, including phenolic compounds, flavonoids, alkaloids and sugars. In particular, *T. sebifera* leaves are rich in a variety of phenolic compounds, such as gallic acid, kaempferol, quercetin, etc., which have potential applications in food and medicine [9,10]. *T. sebifera* leaves have been valued as a source of therapeutic compounds [7], and the potential demand for *T. sebifera* leaves and their products is higher. However, it remains a challenge to sustainably extract and characterize these compounds from leaf wastes. At present, the application value of phenolic compounds in pharmacology, functional foods, food processing and preservation, and cosmetics is increasing [11,12].

Therefore, it has become particularly important to develop effective extraction techniques to recover these valuable compounds from natural resources. A large number of studies have reported on the extraction of phenolic compounds from plant materials and their analytical techniques [13]. Prior to optimizing the extraction process, identification of the major variables is essential to achieve maximum response, optimum time, energy and solvent consumption, and to design a low-cost, high-margin extraction process. Drying is a common processing step in the treatment of extracts that not only improves the nutritional value of the dry matter through concentration, but also facilitates storage and transportation [14]. At present, there are a variety of drying methods available for application, such as hot air drying, vacuum drying and vacuum freeze drying, etc. These drying methods have been widely used in the concentration and drying of a variety of extracts, and each technology has its own specific applicability and effect [15,16,17]. Thus, it is still necessary to choose a suitable drying method to dry the extract to maintain its good biological activity.

Leaf waste contains a variety of bioactive components, and can be utilized to enhance the utilization value of natural resources and reduce the burden of environmental pollution [18,19]. In particular, phenolic compounds extracted from leaf waste have attracted attention for their multiple biological functions such as antioxidant, antitumor, immunomodulatory, antibacterial, and hypolipidemic activity [20]. However, the extraction, activity evaluation and characterization of phenolic compounds from underutilized wastes such as *T. sebifera* leaves have not been sufficiently studied. Therefore, the aim of this study was to select the optimum operating conditions for the extraction of phenolic compounds from *T. sebifera* leaves using Response Surface Methodology (RSM) and Box–Behnken design (BBD) modeling. The effects of different parameters (ethanol content, extraction time, liquid–solid ratio and extraction temperature) and their interactions on the extraction rate were analyzed and investigated. Phenolics compounds of extracts were quantified and correlated using high-performance liquid chromatography (HPLC), and the effects of hot air drying (HD), vacuum drying (VD), and freeze drying (FD) on the antioxidant activity and characterization of *T. sebifera* leaf extract (TSLE) were evaluated by in vitro antioxidant assays (ABTS and DPPH radical scavenging activities), FT-IR and SEM. The results of this study not only provide the optimal process parameters for the extraction of phenolic compounds from *T. sebifera* leaves but also lay the foundation for drying these phenolic antioxidants, thereby maximizing the economic value of *T. sebifera* and avoiding resource wastage. Through these studies, we aim to promote the comprehensive utilization of *T. sebifera* leaves, reduce environmental pollution, and provide valuable data and references for related industries.

## 2. Results and Discussion

### 2.1. Effects of Extraction Parameters on the Extraction Yield

**Figure 1:**
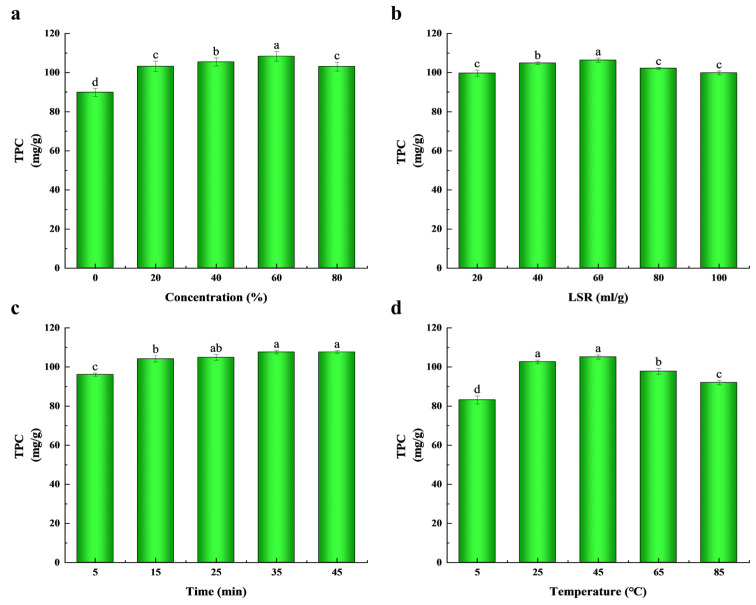
TSP extraction yield was affected by ethanol content (**a**), liquid–solid ratio (LSR) (**b**), extraction time (**c**) and extraction temperature (**d**). Data are expressed as mean ± standard deviation (*n* = 3). Different lowercase letters (a–d) in the graphs indicated statistically significant differences (*p* < 0.05).

As shown in Figure 1, there were significant differences in the TPC extracted from *T. sebifera* leaves under the four extraction parameters (*p* < 0.05). Compared to the extraction with single solvents, the mixture of ethanol and water was more effective in extracting phenolic compounds [21]. In the aqueous cosolvent system, the variation in the solvent concentration could influence the distinct release ability of bioactive compounds from plant tissues, but the extraction yields from different samples exhibited significant differences [22]. As shown in Figure 1a, the TPC gradually increased with the rising ethanol concentration up to 60%, and reached the highest value of 108.9 ± 1.85 mg/g. However, the yield reduced when the concentration exceeded 80%. Ethanol usually increases the solubility of phenolic compounds, while water helps to release them from the sample matrix [23]. In related studies, Sun et al. (2011) found that the maximum amount of TPC could be extracted from *Ilex kudingcha* using 70% ethanol concentration [23]. Similarly, studies by Xu et al. (2017) and Khan et al. (2010) showed that 60% and 80% ethanol concentrations were effective in extracting polyphenols from *Limonium sinuatum* flowers and *Citrus sinensis* peels, respectively [24,25]. This effect may be attributed to the increased solubility of phenolic compounds with increasing ethanol concentration, as non-covalent interactions such as hydrogen bonding and hydrophobic interactions are weakened. However, higher ethanol concentration can reduce the extraction efficiency of phenolic compounds. In addition, interactions between ethanol, water and polyphenols may also affect the extraction process [26]. Therefore, to optimize the extraction process, an ethanol concentration range of 20–80% was selected for the next experiments.

The extraction yield of phenolic compounds in different materials is considerably impacted by the liquid–solid ratio (LSR). As exhibited in Figure 1b, the LSR showed significant positive effects on TPC from 20 to 60, and the maximum TPC of 106.4 ± 1.11 mg/g was found at the LSR of 60. However, a decline appeared as the LSR increased from 60 to 80. This was in agreement with Prakash Maran et al.’s study (2017), in which the yield of polyphenols increased gradually as the LSR increased from 1:10 to 1:20 [27]. The dissolution of phenolic compounds from the internal tissues of the material improved significantly with an increase in the liquid–solid ratio (LSR), which may be related to the effect of the LSR. Usually, an increase in the liquid–solid ratio leads to an increase in the concentration difference, which enhances the mass transfer process and promotes solute dissolution [28]. Nevertheless, the diffusion distance of phenolic compounds within the tissue increases, which results in the enhancement of the extraction rate becoming less significant when the LSR continues to increase. This phenomenon can be explained by the fact that once dissolution reaches equilibrium, further increases in the LSR will not improve the extraction efficiency [29]. This effect was weakened with a higher LSR because the low mass transfer mainly depended on the amount of materials [30]. Therefore, the selected LSR of 50 g/mL as the center point of the BBD experiment also reduces the amount of solvent and saves costs.

Extraction time is an important factor that must be taken into account in the phytoextraction process, as it can help save time, cost and energy [31]. As exhibited in Figure 1c, the TPC showed time-dependent effects from 5 min to 35 min, and the maximum TPC was 107.6 ± 0.86 mg/g under the condition of 35 min. There was no significant difference in TPC between 25 min and 35 min, but the TPC decreased with the increase in extraction time. Although extending the extraction time can enhance the yield of bioactive com-pounds by facilitating their more complete diffusion from the plant material into the solvent [32], excessively long extraction periods can lead to increased energy consumption and costs [28,31]. Furthermore, prolonged extraction may result in elevated temperatures, which can heighten the risk of degradation of phenolic compounds, and the evaporation of the solvent can also diminish the efficiency of mass transfer [33]. Consequently, considering these factors, 25 min was identified as the optimal extraction time for further research.

The heat transfer process is directly sped up by the extraction temperature, which in turn enhances the extraction rate by utilizing thermal energy to disrupt the cellular structures and to reduce both viscosity and surface tension [28]. Thus, extraction temperature significantly improves the release rate of bioactive compounds in solution from plant materials [34]. As exhibited in Figure 1d, within 5–85 °C, the TPC substantially increased up to 45 °C, and reached a maximum of 105.2 ± 1.06 mg/g. And then, the values decreased significantly when the extraction temperature was over 45 °C. This indicated that the extraction temperature exerted positive influences on the extraction yield of the phenolic compounds. However, a higher temperature can result in the degradation of some thermosensitive phenolic compounds, and thus reduce the extraction efficiency of phenolic compounds [35,36]. Moreover, when the temperature overtook the boiling point of ethanol, the evaporation rate of ethanol was accelerated, which also led to adverse effects on the extraction yield [28]. Consequently, 45 °C was chosen as the best extraction temperature.

### 2.2. Model Fitting and Analysis

Based on the analysis of single-factor experiments, a Box–Behnken design with four factors at three levels (response surface methodology) was employed, using the polyphenol extraction yield as the response value. Table 1 presents a total of 27 experimental combinations. The results were subjected to multiple regression fitting using JMP™ Pro 16, and the relationship between the TSP extraction rate and the various variables is expressed in Equation (1):

Y = 109.72 − 9.67X_1_ + 1.95X_2_ + 3.55X_4_ − 3.48X_1_X_2_ − 5.63X_2_X_4_ − 14.04X_1_^2^ − 10.83X_2_^2^ − 5.5X_3_^2^ − 4.91X_4_^2^, (1)

where Y is the predicted value of the TSP extraction yield. X_1_, X_2_, X_3_, and X_4_ are the coded values of the above independent variables, respectively. Based on the F value (Table 2), the influence order of extraction parameters on TPC was X_1_ (solvent concentration) > X_4_ (extraction temperature) > X_2_ (LSR) > X_3_ (extraction time), which indicated that ethanol concentration was the key factor influencing the extraction yield of TSP. 

To assess the significance of these factors and their interactions in the model, the data were analyzed using ANOVA. Table 2 shows the highly correlated plausible predictions (R^2^ = 0.9707) between the predicted and experimental values [37]. The adjusted coefficient of determination was AdjR^2^ = 0.9366, indicating that the model can explain 93.66% of the response value changes, with a high degree of fitting. The Model term is extremely significant (*p* < 0.001), and the Lack of Fit term is not significant (*p* > 0.05), indicating that the model equation is suitable for estimating the TPC extraction yield from *T. sebifera* leaves. Figure 1 demonstrates the rationality of the single-factor experiments, in which X_1_, X_2_, X_4_, X_1_X_2_, X_2_X_4_, X_1_^2^, X_2_^2^, X_3_^2^ and X_4_^2^ have significant effects on the TSP yield (*p* < 0.05).

**Figure 2:**
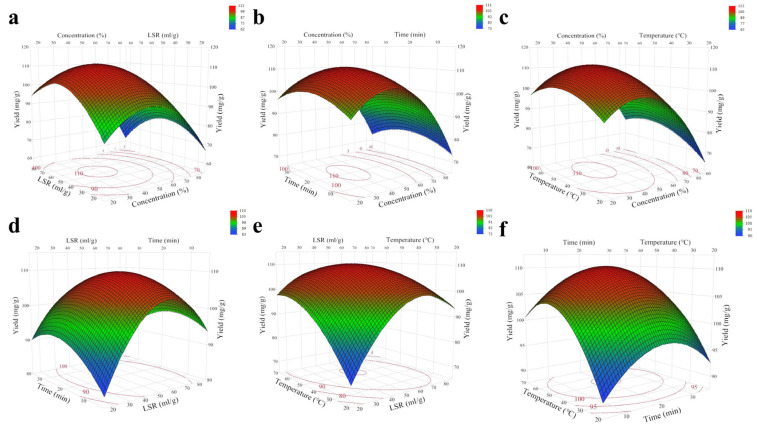
Response surface plots and contour plots for the interactions of various factors on the TSP yield. (**a**) Ethanol concentration and liquid–solid ratio, (**b**) ethanol concentration and extraction time, (**c**), ethanol concentration and extraction temperature, (**d**) liquid–solid ratio and extraction time, (**e**) liquid–solid ratio and extraction temperature and (**f**) extraction temperature and extraction time.

In addition, three-dimensional (3D) response surfaces representing the relationship between TPC and extraction parameters were analyzed using RSM. Figure 2 depicts three-dimensional (3D) surface plots that reveal the impact of extrusion parameters and the interaction between any two factors on the TSP yield. In the three-dimensional surface plot, the steeper the surface, the more significant the interaction between the variables [38]. The contour plot, which is the bottom projection of the response surface, represents the interaction between two factors. If the contour lines tend to be elliptical, it indicates a significant interaction between these two factors; conversely, if the contour lines tend towards a circular shape, the interaction between these factors is not significant [38]. The increase in the four factors results in an upward trend in the TSP yield, but once these factors exceed certain values, their impact on the TSP yield begins to decline.

### 2.3. Verification of Optimal Extraction Conditions and Extraction Yield

Based on Figure 2, the feasibility of the experiment and the parameters that predict the applicability of the maximum yield model equation can be derived as follows: the ethanol concentration was 39.8%, the liquid–solid ratio was 52.1 mL/g, the extraction time was 20.2 min, the extraction temperature was 50.6 °C and the predicted extraction rate of TSP was 111.94 mg/g. Validation experiments were carried out on the basis of the optimal parameters mentioned above, and the results showed that the actual extraction rate of TSP was 111.46 mg/g, which showed a smaller deviation from the above predicted value (RSD = 0.43%). Thus, the model has good fidelity and reproducibility.

### 2.4. Composition Analysis of Phenolic Compounds

Table 3 shows the data of regression equation, linear range, retention time (Rt) and correlation coefficient (R^2^). From the table, the linear ranges and retention times of the eight phenolic compounds were significantly different. Specifically, gallic acid had an R^2^ of 0.9998, while the other seven phenolic compounds had an R^2^ of 0.9999. These high R^2^ values indicate that the analytical method used has a good linear relationship and is therefore suitable for the quantitative analysis of these phenolic compounds. Twelve phenolic compounds were quantitatively analyzed from the TSP samples obtained under optimal conditions, where quercitrin, rutin, cianidanol and kaempferitrin were not detected, and the results of the eight phenolic substances detected are shown in Table 3. The results showed that the highest content of tannins in these samples was 13,988.1 ± 531.5 μg/g and the lowest content of kaempferol was 51.1 ± 0.77 μg/g. The content of phenolic compounds was determined as follows: tannins > isoquercitrin > ellagic acid > ethyl gallate > hyperoside > gallic acid > quercetin > kaempferol. Previous studies on the phenolic compounds of *T. sebifera* leaves have been more qualitative and less quantitative, and the results of four of these phenolic substances have been reported in previous studies [39]. These differences may be due to the great variability of the same species which exists in terms of processing conditions, harvest season, growing and storage time and geographic location [40]. Thus, further studies with samples obtained at different processing conditions and harvest seasons are still needed and can be regarded as an important contribution to the reasonable utilization of these leaves’ waste and, simultaneously, add economic value to this species.

### 2.5. In Vitro Antioxidant Activity

**Figure 3:**
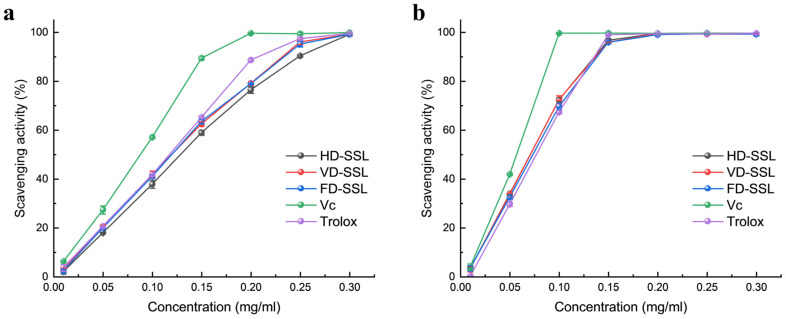
Scavenging activity of the phenolic compound extracts from *T. sebifera* leaves. (**a**) DPPH scavenging activity. (**b**) ABTS scavenging activity. Each value is the mean ± SD of triplicate measurements.

Previous reports have indicated that phenolic compounds have high free radical scavenging activities, and play key roles in scavenging ROS [41]. Due to the differences in the types and contents of the different phenolic compounds, their antioxidant activity also differs. DPPH and ABTS scavenging activity are commonly used methods for evaluating the antioxidant scavenging activity of various bioactive compounds in animal and plant extracts [42,43]. In the present study, the variations in the DPPH scavenging capacity at different concentrations of leaf extracts are shown in Figure 3a, and the scavenging rates of HD-TSLE, VD-TSLE and FD-TSLE exhibited more than 50% at a concentration of 0.15 mg/mL. It is noteworthy that VD-TSLE and FD-TSLE exhibited the most potent DPPH scavenging capacity and were significantly higher than the HD-TSLE. At a concentration of 0.25 mg/mL, the DPPH scavenging capacity of HD-TSLE, VD-TSLE and FD-TSLE exceeded more than 90%, and exhibited further enhancement up to 0.3 mg/mL. The present study showed that the IC50 values of HD-TSLE, VD-TSLE and FD-TSLE were 159.1 ± 3.5 µg/mL, 138.2 ± 5.2 µg/mL and 135.5 ± 1.3 µg/mL, respectively. As shown in Figure 3b, the ABTS scavenging capacity of HD-TSLE, VD-TSLE and FD-TSLE was exhibited in a linear concentration-dependent manner within a certain range. The values of ABTS scavenging capacity, at a concentration of 0.1 mg/mL HD-TSLE, VD-TSLE and FD-TSLE, were higher than those of Trolox. The values of IC50 for HD-TSLE, VD-TSLE, and FD-TSLE were 73.7 ± 2.2 µg/mL, 73.5 ± 4.7 µg/mL, and 74.3 ± 0.9 µg/mL, respectively, which showed no significant differences. These results showed that TSLE had remarkable influences on scavenging DPPH and ABTS free radicals, which was directly associated with TSLE contents and composition.

### 2.6. Correlation Analysis

**Figure 4:**
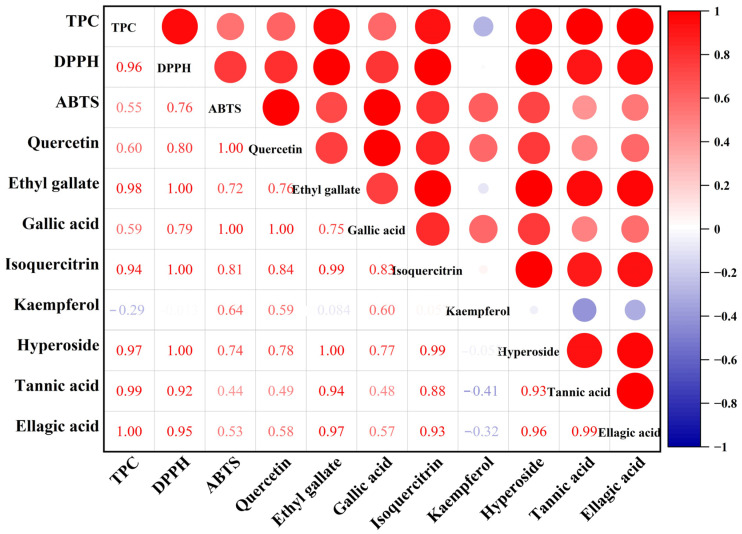
Correlation heat map analysis of TPC, quercetin, ethyl gallate, gallic acid, isoquercitrin, kaempferol, hyperoside, tannic acid, ellagic acid and in vitro antioxidant activities.

The correlation heat map analysis results (Figure 4) showed that the two in vitro antioxidant evaluation methods were positively correlated with TPC, quercetin, ethyl gallate, gallic acid, isoquercitrin, hyperoside, tannic acid and ellagic acid, and most of the correlation coefficients were in the range of 0.53 and 0.95. Therefore, the DPPH and ABTS methods can directly show the in vitro antioxidant capacity of *T. sebifera* leaves. It is noteworthy that ethyl gallate, isoquercitrin, hyperoside, tannic acid, and ellagic acid may contribute more to the DPPH scavenging rate of *T. sebifera* leaves than the other chemical components. In addition, kaempferol, tannic acid and ellagic acid showed a weak positive correlation with the ABTS scavenging capacity of *T. sebifera* leaves, which may contribute to the antioxidant capacity. *T. sebifera* leaves are rich in natural antioxidants [44] (e.g., polyphenols) that play an important role in antioxidant activity. The content of endogenous natural antioxidant actives in *T. sebifera* leaves varied depending on the extraction method and the affinity for scavenging free radicals, leading to significant differences in the antioxidant capacity of *T. sebifera* leaves.

### 2.7. Analysis of SEM

**Figure 5:**
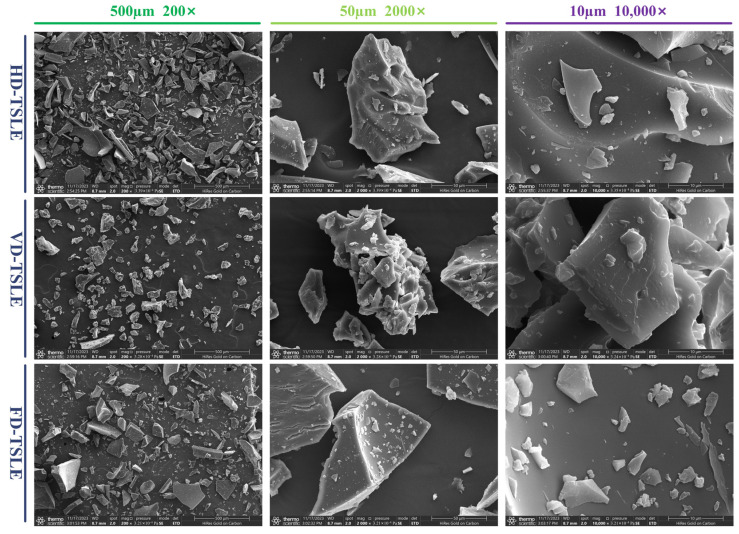
SEM analysis of HD-TSL, VD-TSL and FD-TSL.

Scanning electron microscopy (SEM) is a common method for observing the surface morphology of coarse matter in the micron and submicron range [45]. The microscopic morphology of HD-TSLE, VD-TSLE and FD-TSLE at different magnifications (×200, ×2000, and ×10,000) is shown in Figure 5, and significant differences were found. It can be seen that HD-TSLE has overall sharp and long strip-shaped particles with larger surface attachments, VD-TSLE has an overall rounded shape with more surface attachments and FD-TSLE is overall square with many grooves on the surface. These structural differences affect the antioxidant activity of the phenolic compound extracts from *T. sebifera* leaves (Figure 3). The structure of particles can impact the wettability and solubility of extracts, which are key indexes to the development of instant soluble samples [46]. Therefore, compared with HD-TSLE, the minimum particle size and loose structure of VD-TSLE are favorable for the development of instant soluble products, and the lower processing temperature of FD-TSLE is favorable for maintaining better antioxidant activity. The present study of different drying pretreatments to obtain the phenolic compound extracts provides practical information for drying pretreatments, which will be convenient for commercial applications.

### 2.8. FTIR Analysis

**Figure 6:**
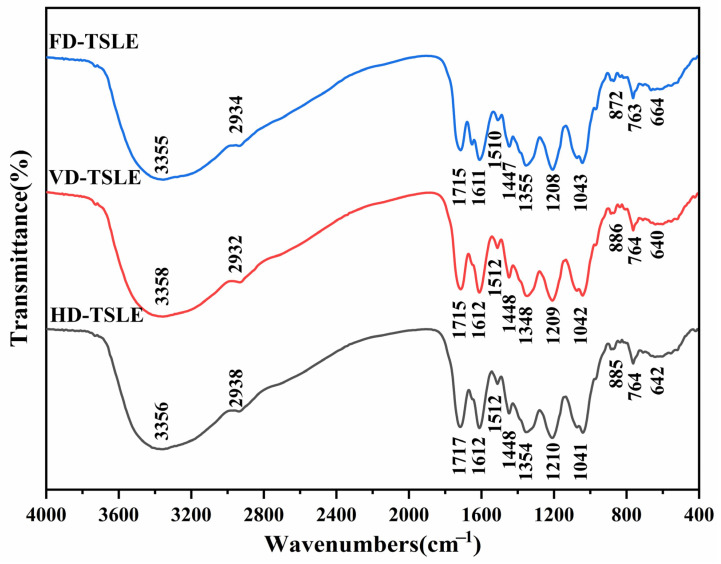
FTIR analysis of HD-TSL, VD-TSL and FD-TSL.

FT-IR spectroscopy provides an efficient tool for characterizing the functional groups of bioactive compounds, such as phenolic compounds, protein and carbohydrates [47,48]. The FT-IR spectrum of HD-TSLE, VD-TSLE and FD-TSLE are depicted in Figure 6, and there were minor visible differences among these samples prepared by different drying methods. The broad band at approximately 3358 cm^−1^ was attributed to the -OH stretching vibration, while the characteristic peak at around 2932 cm^−1^ is responsible for the weaker C-H stretching vibration and variable angle vibrations. In addition, the absorption peaks between 1715 cm^−1^ and 1200 cm^−1^ are characterized by the stretching vibration of the esterified carboxyl group (-COOR), the asymmetric and symmetric stretching of the carboxylic anion group (-COO-), and the bending of C-O and C-H. The characteristic absorption peaks at around 1612 and 1448 cm^−1^ are carboxyl symmetric and asymmetric stretching vibrations. The absorption signals at 1348 and 1209 cm^−1^ are attributed to the C-H variable angle vibration and C-O stretching vibration. The characteristic absorption bands between 950 and 1200 cm^−1^ indicate the existence of C-O-C and C-O-H bonds. The absorption peak at around 886 cm^−1^ may be caused by the C-O-P stretching vibration [43,49,50]. Based on FT-IR analysis, the phenolic compounds from *T. sebifera* leaves treated with three drying methods had similar backbones and chemical groups, showing drying methods did not destroy the preliminary structures of the phenolic compound from *T. sebifera* leaves.

## 3. Materials and Methods

### 3.1. Plant Materials and Reagents

The sample site was located at Jing-shan village, Yong-an town, Shuangliu district, Chengdu, China, at the latitude 30°365954′ N, longitude 104°004114′ W and altitude 411–425 m. The leaves were harvested in July 2023 from five *T. sebifera* plants with height of 5–7 m. After being collected, the leaves were dried at 65 °C, powdered in an electric grinder (FW80, Taisite, Tianjin, China), and then sieved through 60 meshes. These samples were then dried in a ventilated oven at 60 °C for 24 h and stored at room temperature for further use.

Gallic acid, DPPH (1,1-diphenyl-2-picrylhydrazyl), Folin–Ciocalteu’s phenol reagent, ABTS (2,2′-azino-bis (3ethylbenzothiazoline-6-sulfonic acid)), Vitamin C (L-ascorbic acid), and Trolox (6-hydroxy-2,5,7,8-tetramethylchroman-2-carboxylic acid) were purchased from Macklin Company (Shanghai, China). Potassium persulfate (K_2_S_2_O_4_), and sodium bicarbonate (Na_2_CO_3_) were purchased from Xilong Science Co., Ltd. (Chengdu, China). Phenolic acid standards were purchased from Sigma-Aldrich (St. Louis, MO, USA). All the chemicals were of analytical and HPLC grade.

### 3.2. Single-Factor Experiments

As shown in Table 4, a single-factor experimental design was established to explore the effects of ethanol concentration, liquid–solid ratio, extraction time and extraction temperature on total phenol content (TPC). A total of 1.0 g of *T. sebifera* leaf powder was weighed into a conical flask, the rest of the conditions are shown in Table 4, and it was placed on a magnetic thermostatic heating stirrer for extraction (SCI1280-Pro, Scilogex, Pleasanton, CA, USA); the rotational speed was fixed at 600 rpm, and after the extraction was finished, it was centrifuged for 5 min at 4 °C with a rotational speed of 8000 rpm, and then the supernatant was taken up and stored at −20 °C for spare use.

### 3.3. Response Surface Design

According to the results of the single-factor experiments, 1.0 g of *T. sebifera* leaf powder was also weighed in a conical flask, and the ethanol content, liquid–solid ratio, extraction time and extraction temperature were selected as the independent variables (Table 5). The extraction processes were performed on a magnetic thermostatic heating stirrer at a speed of 600 rpm. A Box–Behnken design (BBD) with four factors and three levels was used to statistically optimize the extraction process. The extraction yields of phenolic compounds from *T. sebifera* leaves were the dependent variable. The experimental design and the actual levels of each factor are shown in Table 5. The significance of the model was assessed using analysis of variance (ANOVA). The accuracy and heritability of the polynomial model can be assessed by the coefficient of determination R^2^ and the adjusted coefficient of determination adjR^2^. In addition, several confirmatory experiments were performed to verify the validity of the statistical experimental strategies.

### 3.4. Determination of Phenolic Compound Content

The phenolic compound contents were analyzed using Folin–Ciocalteu’s reagent with slight modifications [51]. In brief, each sample of 400 μL was combined with 1.2 mL of 7.5% Na_2_CO_3_, 2 mL of distilled water and 400 μL of Folin–Ciocalteau reagent. The mixed solutions stayed for 60 min in a dark place at room temperature, and their absorbance was recorded at a wavelength of 765 nm using a UV spectrophotometer using gallic acid as the standard. The standard curve was drawn with the concentration of gallic acid standard solution as the X axis and the absorbance value as the Y axis. The result was expressed in milligrams of gallic acid equivalent (GAE) per gram of dry weight (mg GAE/g dw).

### 3.5. Validation of the Model

According to the response model, verification experiments of phenolic compound content were performed using the optimal extraction conditions of the BBD to check the accuracy of the response model. All reactions were measured under optimized extraction conditions. The experimental values were compared with the predicted values based on CV% to determine the effectiveness of the model.

### 3.6. High-Performance Liquid Chromatography (HPLC) Analysis

High-performance liquid chromatography (HPLC) and a UV-DAD diode array detection system (Agilent, Clara, CA, USA) were used to analyze the composition of phenolic compounds. The chromatographs, columns, column temperatures, flow rates, injection volumes, mobile phases and wavelengths used for the determination of the 12 phenolic compounds are shown in Table S1. The HPLC chromatograms of the authentic standards and *T. sebifera* leaf extract under the optimum conditions are shown in Figures S1–S5. Quercetin, ethyl gallate, gallic acid, isoquercetin, quercetin, kaempferol, rutin, hyperoside, tannic acid, cianidanol, ellagic acid, and kaempferitrin were accurately weighed as the standards, and then dissolved in methanol, and configured into five or six standard solutions with different mass concentrations. After derivatization, the peak areas of each standard solution were sequentially detected according to the above chromatographic conditions, and their standard curves were calculated by taking the peak area as the vertical coordinate and the concentration as the horizontal coordinate, and the linear ranges and correlation coefficients are shown in Table 3. The precision test procedure is as follows: take the standard solution, repeat the injection 6 times, and record the peak area. The stability experiment procedure is as follows: take the test solution at 0, 2, 4, 6, 8, 10 h for injection determination. The standard addition recovery test procedure is as follows: take six prepared sample solutions, take 0.5 mL, add 0.5 mL of mixed control solution (the authentic standards for 12 phenolic compounds), perform sample determination, and obtain the recovery results. These results are shown in Table S2. Finally, the results were expressed as ug per gram of dried *T. sebifera* leaf powder (μg/g).

### 3.7. Leaf Extract Preparation and Drying Process

Under optimal extraction conditions, 10 g of leaves was weighed for extraction. After extraction, the supernatant was collected and concentrated using a rotary evaporator equipped with a water circulation vacuum pump (RE-52A, Shanghai Yarong Instruments Co., Ltd., Shanghai, China). The concentrated sample was then subjected to drying processes, including hot air drying (HD), vacuum drying (VD) and freeze drying (FD). Hot air drying was performed in an electrically heated blast dryer (101-2-BS, Beijing Yong Guangming Medical Instrument Co., Ltd., Beijing, China) at a temperature of 50 °C. Vacuum drying was performed in a vacuum drying oven (DZF, Shanghai Longyue Instruments Equipment Co., Ltd., Shanghai, China) at 50 °C. Freeze drying was performed in a vacuum freeze drying machine (SCIENTZ-12N, Ningbo Scientz Biotechnology Co., Ltd., Ningbo, China) at −40 °C. After drying, these extracts were stored in airtight containers at 4 °C until use. The *T. sebifera* leaf extracts obtained by hot air drying, vacuum drying and freeze drying were labeled as HD-TSLE, VD-TSLE and FD-TSLE, respectively. Finally, the dried samples were dissolved under the same conditions using the ethanol concentration from the best extraction conditions (39.8%) to assess their antioxidant activity.

### 3.8. In Vitro Antioxidant Activity

#### 3.8.1. DPPH Radical Scavenging Capacity

DPPH radical scavenging capacity was determined according to a previous method [52]. In brief, 100 µL of the extracts was blended with 900 µL DPPH solution (0.06 mM), and then were left in the dark for 30 min at room temperature. The reaction mixtures were well shaken, and the absorbance was read at 517 nm using a UV–visible spectrophotometer. DPPH radical scavenging rate (%) = (1 − A/A0) × 100%. Distilled water was used as a blank control (A0); VC (vitamin C) and Trolox were used as a positive control.

#### 3.8.2. ABTS Radical Scavenging Capacity

The ABTS radical scavenging capacity assay was evaluated according to the method of Ren et al. (2023) [53]. In brief, ABTS solution (7.0 mM) and potassium persulfate solution (2.45 mM) were mixed in equal quantities, and left in the dark for 16 h at room temperature. A total 200 µL of the extracts and 800 µL ABTS solution were blended, and kept in the dark for 6 min, and then the absorbance of these mixtures was recorded at 734 nm. ABTS radical scavenging rate (%) = (1 − A/A0) × 100%. Distilled water was used as a blank control (A0); VC (vitamin C) and Trolox were used as a positive control.

### 3.9. Scanning Electron Microscopy (SEM) Analysis

The microstructures of HD-TSLE, VD-TSLE and FD-TSLE powders were observed according to the method described by Wang et al. (2023) [54], with slight modifications. The dried samples were pressed, gold sprayed and examined using a scanning electron microscope analyzer (SU8010, Hitachi, Tokyo, Japan). Representative areas were selected and photographed, and the images were compared at 200×, 2000× and 10,000× magnifications.

### 3.10. Fourier Transform Infrared Spectroscopy (FTIR)

HD-TSLE, VD-TSLE and FD-TSLE powders (1 mg) were evenly mixed with dried KBr (200 mg), ground, pressed into thin tablets, and scanned in a waveband range between 4000 and 400 cm^−1^ using a Fourier transform infrared spectrometer (IS50, Thermo Fisher Scientific, Waltham, MA, USA).

### 3.11. Statistical Analysis

All tests were performed in triplicate, and data were expressed as mean ± standard deviations (SD). Experimental results were analyzed with one-way analysis of variance (ANOVA), performed at a significance level of 0.05. The entire statistics-related response surface methodology and experimental design were carried out using JMP™ Pro 16. The graphs were plotted using Origin 2023 (Origin Lab, Northampton, MA, USA).

## 4. Conclusions

Although studies have conducted a lot of research on the extraction of phenolic compounds from leaves and their components, this is the first time a simple and easy-to-scale-up process for obtaining high antioxidant phenolic compounds from *T. sebifera* leaves has been established. In this study, RSM was used to successfully evaluate the effects of extraction factors and their interactions on extraction amount, antioxidant activity and phenolic compound composition. The optimal extraction parameters of ethanol content 39.8%, liquid–solid ratio 52.1, extraction time 20.1 min and extraction temperature 50.6 °C were selected, and the highest extraction yield was 111.46 mg/g, with an error of less than 0.05%. The results showed that the measured and theoretical values had a good fit, and the optimized process conditions were suitable for the extraction of phenolic compounds from *T. sebifera* leaves, which could be used in pilot scale and large-scale production. HPLC analysis showed that quercetin, ethyl gallate, gallic acid, isoquercitrin, kaempferol, hyperoside, tannic acid and ellagic acid were the main components of the antioxidant capacity of *T. sebifera* leaf extract. Based on the correction analysis, tannic acid, isoquercitrin and ellagic acid are the major contributors to the antioxidant activity. In addition, in vitro antioxidant, FT-IR and SEM results confirmed that the phenolic antioxidant activity of *T. sebifera* leaf extract produced by VD and FD was significantly higher than that of the extract obtained by HD. Therefore, VD and FD are better methods to prepare phenolic antioxidants from *T. sebifera* leaf extract, which has practical application value. The present study can provide a technical reserve for the extraction and drying of polyphenol antioxidants from *T. sebifera* leaves, and provide a practical scheme and basis for further refining these bioactive compounds and developing nutritional health products and cosmetic raw materials.

## Figures and Tables

**Table 1:** Box–Behnken design (BBD) and resultant responses.

Run	X_1_: Ethanol Concentration (%)	X_2_: LSR (mL/g)	X_3_: Time (min)	X_4_: Temperature (°C)	TPC (mg/g) *
1	−1(20)	−1(20)	0(20)	0(45)	88.34
2	−1(20)	0(50)	0(20)	−1(25)	94.98
3	−1(20)	0(50)	−1(5)	0(45)	99.89
4	−1(20)	0(50)	1(35)	0(45)	104.93
5	−1(20)	0(50)	0(20)	1(65)	101.75
6	−1(20)	1(80)	0(20)	0(45)	99.65
7	0(50)	−1(20)	0(20)	−1(25)	84.98
8	0(50)	−1(20)	−1(5)	0(45)	86.98
9	0(50)	−1(20)	1(35)	0(45)	91.34
10	0(50)	−1(20)	0(20)	1(65)	103.33
11	0(50)	0(50)	−1(5)	−1(25)	97.04
12	0(50)	0(50)	1(35)	−1(25)	97.12
13	0(50)	0(50)	0(20)	0(45)	111.02
14	0(50)	0(50)	0(20)	0(45)	109.98
15	0(50)	0(50)	0(20)	0(45)	108.16
16	0(50)	0(50)	−1(5)	1(65)	103.33
17	0(50)	0(50)	1(35)	1(65)	100.98
18	0(50)	1(80)	0(20)	−1(25)	98.67
19	0(50)	1(80)	−1(5)	0(45)	97.12
20	0(50)	1(80)	1(35)	0(45)	91.03
21	0(50)	1(80)	0(20)	1(65)	94.51
22	1(80)	−1(20)	0(20)	0(45)	77.68
23	1(80)	0(50)	0(20)	−1(25)	73.93
24	1(80)	0(50)	−1(5)	0(45)	78.82
25	1(80)	0(50)	1(35)	0(45)	82.54
26	1(80)	0(50)	0(20)	1(65)	85.46
27	1(80)	1(80)	0(20)	0(45)	75.09

* Mean of triplicate determination.

**Table 2:** Variance analysis of the fitted second-order polynomial models in extraction yield of TSP.

Source	df	Sum of Squares	Mean Square	*F* Value	*p* Value
Model	14	2821.1521	201.511	28.4210	<0.0001 ***
X_1_-Concentration (%)	1	1121.7200	1121.7200	158.2068	<0.0001 ***
X_2_-LSR (mL/g)	1	45.7080	45.7080	6.4466	0.0260 *
X_3_-Time (min)	1	1.8881	1.8881	0.2663	0.6152
X_4_-Temperature (°C)	1	151.5141	151.5141	21.3695	0.0006 ***
X_1_X_2_	1	48.3025	48.3025	6.8126	0.0228 *
X_1_X_3_	1	0.4356	0.4356	0.0614	0.8084
X_1_X_4_	1	27.3006	27.3006	3.8505	0.0733
X_2_X_3_	1	5.6644	5.6644	0.7989	0.3890
X_2_X_4_	1	126.6750	126.6750	17.8662	0.0012 **
X_3_X_4_	1	1.4762	1.4762	0.2082	0.6563
X_1_^2^	1	1051.2528	1051.2528	148.2682	<0.0001 ***
X_2_^2^	1	625.7815	625.7815	88.2599	<0.0001 ***
X_3_^2^	1	163.2210	163.2210	23.0206	0.0004 ***
X_4_^2^	1	128.6857	128.6857	18.1498	0.0011 **
Residual	12	85.0826	7.079		
Lack of Fit	10	80.8914	8.0891	3.8601	0.2232
Pure Error	2	4.1912	2.0956		
Cor Total	12	84.0825			
R^2^	0.9707				
Adj R^2^	0.9366				

Note: *, the difference is significant at 0.05 level (*p* < 0.05). **, the difference is significant at 0.01 level (*p* < 0.01). ***, the difference is significant at 0.001 level (*p* < 0.001)

**Table 3:** Identification of the compositions of *T. sebifera* leaf extracts.

ID	Compounds	Formula	Regression Equation	Linear Range (μg/mL)	R^2^	Rt (min)	Concentrations (μg/g)
1	Quercetin	C_15_H_10_O_7_	y = 7.1294x − 0.1801	0.1–40	0.9999	18.667	177.98 ± 26.03
2	Ethyl gallate	C_9_H_10_O_5_	y = 18.5491x − 1.4280	0.1–100	0.9999	23.121	3469.99 ± 79.19
3	Gallic acid	C_7_H_6_O_5_	y = 22.3831x − 0.6774	0.1–40	0.9998	7.793	651.27 ± 22.73
4	Isoquercitrin	C_21_H_20_O_12_	y = 11.1103x − 4.0684	1–200	0.9999	8.244	9905.99 ± 328.13
5	Kaempferol	C_15_H_10_O_6_	y = 8.8767x − 0.3402	0.4–100	0.9999	20.468	51.11 ± 0.77
6	Hyperoside	C_21_H_20_O_12_	y = 9.3525x − 0.5628	0.4–100	0.9999	7.933	958.33 ± 69.13
7	Tannic acid	C_76_H_52_O_46_	y = 1.4179x − 4.3884	1–1000	0.9999	4.33	13,988.12 ± 531.46
8	Ellagic acid	C_14_H_6_O_8_	y = 46.5936x − 1.4697	0.4–200	0.9999	7.302	5161.07 ± 512.88

**Table 4:** Single-factor experimental design.

Single Factor	Ethanol Concentration (%)	Liquid–Solid Ratio (mL/g)	Time (min)	Temperature (°C)
Ethanol concentration	0,20,40,60,80	40	40	40
Liquid–solid ratio	40	20,40,60,80,100	40	40
Extraction Time	20	20	5,15,25,35,45	20
Temperature	25	25	25	5,25,45,65,85

**Table 5:** Factors and their coding levels in experimental design for RSM.

Factors	Levels		
	−1	0	1
X1: Ethanol concentration (%)	20	50	80
X2: Liquid–solid ratio (mL/g)	20	50	80
X3: Extraction time (min)	5	20	35
X4: Extraction temperature (°C)	25	45	65

## Data Availability

The data presented in this study are available on request from the corresponding author.

## Supplementary Materials

The following supporting information can be downloaded at: https://www.mdpi.com/article/10.3390/molecules29143266/s1, Table S1: The chromatograph, column, column temperature, flow rate, injection volume, mobile phase and wavelength for the determination of 12 phenolic compounds were used. Table S2: HPLC LOD, LOQ, precision, stability and standard addition recovery test for the determination of 12 phenolic compounds. Figure S1: HPLC chromatogram of the authentic standards and *T. sebifera* leaves extract under optimum conditions. Peaks 1 is tannic acid. Figure S2 HPLC chromatogram of the authentic standards and *T. sebifera* leaves extract under optimum conditions. Peaks 2, 3 and 4 are gallic acid, cianidanol and ethyl gallate, respectively. Figure S3: HPLC chromatogram of the authentic standardsand *T. sebifera* leaves extract under optimum conditions. Peaks 5, 6 and 7 are rutin, quercetin and kaempferol, respectively. Figure S4: HPLC chromatogram of the authentic standards and *T. sebifera* leaves extract under optimum conditions. Peaks 8 is ellagic acid. Figure S5: HPLC chromatogram of the authentic standards and *T. sebifera* leaves extract under optimum conditions. Peaks 9, 10, 11 and 12 are kaempferitrin, hyperoside, isoquercitrin and quercitrin, respectively.

## References

[1] Peng D., Zhou B., Jiang Y., Tan X., Yuan D., Zhang L. (2018). Enhancing Freezing Tolerance of Brassica Napus L. by Overexpression of a Stearoyl-Acyl Carrier Protein Desaturase Gene (SAD) from *Sapium sebiferum* (L.) Roxb. Plant Sci..

[2] Hou J., Mao Y., Su P., Wang D., Chen X., Huang S., Ni J., Zhao W., Wu L. (2020). A High Throughput Plant Regeneration System from Shoot Stems of *Sapium sebiferum* Roxb., a Potential Multipurpose Bioenergy Tree. Ind. Crops Prod..

[3] Wang R., Hanna M.A., Zhou W.-W., Bhadury P.S., Chen Q., Song B.-A., Yang S. (2011). Production and Selected Fuel Properties of Biodiesel from Promising Non-Edible Oils: *Euphorbia lathyris* L., *Sapium sebiferum* L. and *Jatropha curcas* L.. Bioresour. Technol..

[4] Zhou B., Fei W., Yang S., Yang F., Qu G., Tang W., Ou J., Peng D. (2020). Alteration of the Fatty Acid Composition of Brassica Napus L. via Overexpression of Phospholipid: Diacylglycerol Acyltransferase 1 from *Sapium sebiferum* (L.) Roxb. Plant Sci..

[5] Yu Y., Liu Y. (2020). Transcriptomics of Chinese *Sapium sebiferum* (L.) Roxb Seed to Reveal Key Enzymes Involved in Oil Accumulation. Oil Crop Sci..

[6] Ma M., Li X., Liang H., Jiang H., Cui H. (2023). Chemical Constituents Variation of Seed Oil of the Chinese Tallow Tree (*Sapium sebiferum* (L.) Roxb) at Different Harvesting Time. Ind. Crops Prod..

[7] Fu R., Zhang Y., Peng T., Guo Y., Chen F. (2015). Phenolic Composition and Effects on Allergic Contact Dermatitis of Phenolic Extracts *Sapium sebiferum* (L.) Roxb. Leaves. J. Ethnopharmacol..

[8] Fu R., Zhang Y.-T., Guo Y.-R., Huang Q.-L., Peng T., Xu Y., Tang L., Chen F. (2013). Antioxidant and Anti-Inflammatory Activities of the Phenolic Extracts of *Sapium sebiferum* (L.) Roxb. Leaves. J. Ethnopharmacol..

[9] Su S., Shen Q., Wang S., Song G. (2023). Discovery, Disassembly, Depolymerization and Derivatization of Catechyl Lignin in Chinese Tallow Seed Coats. Int. J. Biol. Macromol..

[10] Fu R., Zhang Y., Guo Y., Chen F. (2015). Chemical Composition, Antioxidant and Antimicrobial Activity of Chinese Tallow Tree Leaves. Ind. Crops Prod..

[11] Jiang J., Qian S., Song T., Lu X., Zhan D., Zhang H., Liu J. (2024). Food-Packaging Applications and Mechanism of Polysaccharides and Polyphenols in Multicomponent Protein Complex System: A Review. Int. J. Biol. Macromol..

[12] Montenegro-Landívar M.F., Tapia-Quirós P., Vecino X., Reig M., Valderrama C., Granados M., Cortina J.L., Saurina J. (2021). Polyphenols and Their Potential Role to Fight Viral Diseases: An Overview. Sci. Total Environ..

[13] Gligor O., Mocan A., Moldovan C., Locatelli M., Crișan G., Ferreira I.C.F.R. (2019). Enzyme-Assisted Extractions of Polyphenols—A Comprehensive Review. Trends Food Sci. Technol..

[14] Shang H., Zhou H., Duan M., Li R., Wu H., Lou Y. (2018). Extraction Condition Optimization and Effects of Drying Methods on Physicochemical Properties and Antioxidant Activities of Polysaccharides from Comfrey (*Symphytum officinale* L.) Root. Int. J. Biol. Macromol..

[15] Duarah P., Joardar S., Debnath B., Purkait M.K. (2024). Optimized Extraction of Polyphenols from Tea Factory Waste and Cost-Effective Drying Methods for Sustainable Utilization. Bioresour. Technol. Rep..

[16] Rubel I.A., Iraporda C., Novosad R., Cabrera F.A., Genovese D.B., Manrique G.D. (2018). Inulin Rich Carbohydrates Extraction from Jerusalem Artichoke (*Helianthus tuberosus* L.) Tubers and Application of Different Drying Methods. Food Res. Int..

[17] Sukadeetad K., Nakbanpote W., Heinrich M., Nuengchamnong N. (2018). Effect of Drying Methods and Solvent Extraction on the Phenolic Compounds of *Gynura pseudochina* (L.) DC. Leaf Extracts and Their Anti-Psoriatic Property. Ind. Crops Prod..

[18] Ben Mabrouk A., Putaux J.-L., Boufi S. (2023). Valorization of Olive Leaf Waste as a New Source of Fractions Containing Cellulose Nanomaterials. Ind. Crops Prod..

[19] Danise T., Innangi M., Curcio E., Piccolella S., Fioretto A., Pacifico S. (2021). White Poplar (*Populus alba* L.) Leaf Waste Recovery and Intercropping Outcome on Its Polyphenols. Ind. Crops Prod..

[20] Andreou V., Psarianos M., Dimopoulos G., Tsimogiannis D., Taoukis P. (2020). Effect of Pulsed Electric Fields and High Pressure on Improved Recovery of High-added-value Compounds from Olive Pomace. J. Food Sci..

[21] Metrouh-Amir H., Duarte C.M.M., Maiza F. (2015). Solvent Effect on Total Phenolic Contents, Antioxidant, and Antibacterial Activities of *Matricaria pubescens*. Ind. Crops Prod..

[22] Zhang S., Xie H., Huang J., Chen Q., Li X., Chen X., Liang J., Wang L. (2024). Ultrasound-Assisted Extraction of Polyphenols from Pine Needles (*Pinus elliottii*): Comprehensive Insights from RSM Optimization, Antioxidant Activity, UHPLC-Q-Exactive Orbitrap MS/MS Analysis and Kinetic Model. Ultrason. Sonochem..

[23] Sun Y., Xu W., Zhang W., Hu Q., Zeng X. (2011). Optimizing the Extraction of Phenolic Antioxidants from Kudingcha Made Frrom Ilex Kudingcha C.J. Tseng by Using Response Surface Methodology. Sep. Purif. Technol..

[24] Xu D.-P., Zheng J., Zhou Y., Li Y., Li S., Li H.-B. (2017). Ultrasound-Assisted Extraction of Natural Antioxidants from the Flower of *Limonium sinuatum*: Optimization and Comparison with Conventional Methods. Food Chem..

[25] Khan M.K., Abert-Vian M., Fabiano-Tixier A.-S., Dangles O., Chemat F. (2010). Ultrasound-Assisted Extraction of Polyphenols (Flavanone Glycosides) from Orange (*Citrus sinensis* L.) Peel. Food Chem..

[26] Umego E.C., Barry-Ryan C. (2024). Optimisation of Polyphenol Extraction for the Valorisation of Spent Gin Botanicals. LWT.

[27] Prakash Maran J., Manikandan S., Vigna Nivetha C., Dinesh R. (2017). Ultrasound Assisted Extraction of Bioactive Compounds from *Nephelium Lappaceum* L. Fruit Peel Using Central Composite Face Centered Response Surface Design. Arab. J. Chem..

[28] Vo T.P., Nguyen L.N.H., Le N.P.T., Mai T.P., Nguyen D.Q. (2022). Optimization of the Ultrasonic-Assisted Extraction Process to Obtain Total Phenolic and Flavonoid Compounds from Watermelon (*Citrullus lanatus*) Rind. Curr. Res. Food Sci..

[29] Li J., Chen Z., Shi H., Yu J., Huang G., Huang H. (2023). Ultrasound-Assisted Extraction and Properties of Polysaccharide from *Ginkgo Biloba* Leaves. Ultrason. Sonochem..

[30] Luo S., Zeng C., Luo F., Li M., Feng S., Zhou L., Chen T., Yuan M., Huang Y., Ding C. (2020). Optimization of Ultrasound-Assisted Extraction of Triterpenes from *Bergenia emeiensis* Leaves and Inhibition Effect on the Growth of Hela Cells. J. Appl. Res. Med. Aromat. Plants.

[31] Rao M.V., Sengar A.S., Sunil C.K., Rawson A. (2021). Ultrasonication—A Green Technology Extraction Technique for Spices: A Review. Trends Food Sci. Technol..

[32] Chen X., Jia X., Yang S., Zhang G., Li A., Du P., Liu L., Li C. (2022). Optimization of Ultrasonic-Assisted Extraction of Flavonoids, Polysaccharides, and Eleutherosides from *Acanthopanax senticosus* Using Response Surface Methodology in Development of Health Wine. LWT.

[33] Ciric A., Krajnc B., Heath D., Ogrinc N. (2020). Response Surface Methodology and Artificial Neural Network Approach for the Optimization of Ultrasound-Assisted Extraction of Polyphenols from Garlic. Food Chem. Toxicol..

[34] Chia S.R., Chew K.W., Leong H.Y., Manickam S., Show P.L., Nguyen T.H.P. (2020). Sonoprocessing-Assisted Solvent Extraction for the Recovery of Pigment-Protein Complex from *Spirulina platensis*. Chem. Eng. J..

[35] Nipornram S., Tochampa W., Rattanatraiwong P., Singanusong R. (2018). Optimization of Low Power Ultrasound-Assisted Extraction of Phenolic Compounds from Mandarin (*Citrus reticulata* Blanco Cv. Sainampueng) Peel. Food Chem..

[36] Setyaningsih W., Saputro I.E., Palma M., Barroso C.G. (2016). Pressurized Liquid Extraction of Phenolic Compounds from Rice (*Oryza sativa*) Grains. Food Chem..

[37] Ferreira S.L.C., Bruns R.E., Ferreira H.S., Matos G.D., David J.M., Brandão G.C., da Silva E.G.P., Portugal L.A., dos Reis P.S., Souza A.S. (2007). Box-Behnken Design: An Alternative for the Optimization of Analytical Methods. Anal. Chim. Acta.

[38] Sun Y., Lu J., Li J., Li P., Zhao M., Xia G. (2023). Optimization of Ultrasonic-Assisted Extraction of Polyphenol from Areca Nut (*Areca catechu* L.) Seeds Using Response Surface Methodology and Its Effects on Osteogenic Activity. Ultrason. Sonochem..

[39] He Q., Zhang L., Li T., Li C., Song H., Fan P. (2021). *Genus sapium* (Euphorbiaceae): A Review on Traditional Uses, Phytochemistry, and Pharmacology. J. Ethnopharmacol..

[40] de Lima D.P., dos Santos Pinto Júnior E., de Menezes A.V., de Souza D.A., de São José V.P.B., da Silva B.P., de Almeida A.Q., de Carvalho I.M.M. (2024). Chemical Composition, Minerals Concentration, Total Phenolic Compounds, Flavonoids Content and Antioxidant Capacity in Organic and Conventional Vegetables. Food Res. Int..

[41] Teixeira F., Silva A.M., Sut S., Dall’Acqua S., Ramos O.L., Ribeiro A.B., Ferraz R., Delerue-Matos C., Rodrigues F. (2024). Ultrasound-Assisted Extraction of Bioactive Compounds from Goji Berries: Optimization, Bioactivity, and Intestinal Permeability Assessment. Food Res. Int..

[42] Wang L., Sun Z., Wang L., Tian S. (2024). Optimization of Ultrasonic-Assisted Extraction of Polyphenols from *Salvia deserta* Schang Flowers Based on Response Surface Methodology and Deep Neural Network and Analysis of Its in Vitro Antioxidant Activities. Ind. Crops Prod..

[43] Chen X., He Y., Liu Z., Huang Z., Xu C., Liu Y., Haran Y., Nisar W., Yan S., Li J. (2024). Ultrasound-Assisted Extraction of Polyphenols from Lotus Rhizome Epidermis by Alcohol/Salt-Based Aqueous Two-Phase System: Optimization, Extraction Mechanism and Antioxidant Activities. Food Chem..

[44] Giusti F., Caprioli G., Ricciutelli M., Vittori S., Sagratini G. (2017). Determination of Fourteen Polyphenols in Pulses by High Performance Liquid Chromatography-Diode Array Detection (HPLC-DAD) and Correlation Study with Antioxidant Activity and Colour. Food Chem..

[45] Mahato P.L., Weatherby T., Ewell K., Jha R., Mishra B. (2024). Scanning Electron Microscope-Based Evaluation of Eggshell Quality. Poult. Sci..

[46] Budnimath S.H., Bhuvaneshwari G., Ganiger V.M., Jagadeesh S.L., Goudar G., Patil S.N., Chandrashekar V.M. (2023). Physical, Reconstitution and Phenolic Properties of Instant Drink Mix Prepared with *Moringa Oleifera* Leaf, Raw Banana and Whey Protein Concentrate. Meas. Food.

[47] Lv Y., Cai X., Shi N., Gao H., Zhang Z., Yan M., Li Y. (2024). Emulsification Performance and Stabilization Mechanism of Okra Polysaccharides with Different Structural Properties. Food Hydrocoll..

[48] Oliveira R.N., Mancini M.C., de Oliveira F.C.S., Passos T.M., Quilty B., Thiré R.M.d.S.M., McGuinness G.B. (2016). FTIR Analysis and Quantification of Phenols and Flavonoids of Five Commercially Available Plants Extracts Used in Wound Healing. Matér. Rio J..

[49] Cao Y., Song Z., Dong C., Ni W., Xin K., Yu Q., Han L. (2023). Green Ultrasound-Assisted Natural Deep Eutectic Solvent Extraction of Phenolic Compounds from Waste Broccoli Leaves: Optimization, Identification, Biological Activity, and Structural Characterization. LWT.

[50] Iftikhar M., Zhang H., Iftikhar A., Raza A., Begum N., Tahamina A., Syed H., Khan M., Wang J. (2020). Study on Optimization of Ultrasonic Assisted Extraction of Phenolic Compounds from Rye Bran. LWT.

[51] Zhong X., Zhang S., Wang H., Yang J., Li L., Zhu J., Liu Y. (2022). Ultrasound-Alkaline Combined Extraction Improves the Release of Bound Polyphenols from Pitahaya (*Hylocereus undatus* ‘Foo-Lon’) Peel: Composition, Antioxidant Activities and Enzyme Inhibitory Activity. Ultrason. Sonochem..

[52] Wang X., Liu X., Shi N., Zhang Z., Chen Y., Yan M., Li Y. (2023). Response Surface Methodology Optimization and HPLC-ESI-QTOF-MS/MS Analysis on Ultrasonic-Assisted Extraction of Phenolic Compounds from Okra (*Abelmoschus esculentus*) and Their Antioxidant Activity. Food Chem..

[53] Ren H.-Y., Qian W.-Z., Yi L., Ye Y.-L., Gu T., Gao S., Cao G.-X. (2023). Nutrient Composition and Antioxidant Activity of *Cercis Chinensis* Flower in Response to Different Development Stages. Horticulturae.

[54] Wang A., Wang Y., Kan H., Hao J., Hu Q., Lu B., Liu Y. (2023). Comparison of Different Drying Techniques for *Zanthoxylum Bungeanum* Leaves: Changes in Color, Microstructure, Antioxidant Capacities, and Volatile Components. LWT.

