# Supplementary material for: Extraction Optimization of Phenolic Compounds from Triadica sebifera Leaves: Identification, Characterization and Antioxidant Activity
Source: Molecules. 2024 Jul 10;29(14):3266. doi: 10.3390/molecules29143266 (PMC11278767; doi:10.3390/molecules29143266)
Supplement: Supplementary file 1 [file molecules-29-03266-s001.zip › molecules-3063127-supplementary.pdf]

Supplementary File

Table S1. The chromatograph, column, column temperature, flow rate, injection volume, mobile phase and wavelength for the determination of 12 phenolic compounds were used.

**metric name**	**Chromatograph**	**chromatographic column**	**Column temperature**	**flow rate (mL/min)**	**Injection volume**	**mobile phase （V/V）**	**wavelength （nm）**
Quercetin	Agilent 1100 high performance liquid chromatograph	Compass C18 (2) reversed phase chromatographic column (250 mm*4.6 mm, 5 μ m)	30 ºC	1.0	5 μL	0.1 % phosphoric acid in water：methanol=35:65	360
Ethyl gallate				0.8		0.1 % phosphoric acid in water：methanol=57:43	280
Gallic acid				0.8		0.1 % phosphoric acid in water：methanol=90:10	280
Isoquercitrin				1.0		0.1 % phosphoric acid in water：acetonitrile =80:20	360
Quercitrin				1.0		0.1 % phosphoric acid in water：acetonitrile =80:20	360
Kaempferol				1.0		0.1 % phosphoric acid in water：methanol=35:65	360
Rutin				1.0		0.1 % phosphoric acid in water：methanol=70:30	360
Hyperoside				1.0		0.1 % phosphoric acid in water：acetonitrile =80:20	360
Tannic acid				1.0		0.1 % phosphoric acid in water：methanol=80:20	275
Cianidanol				0.8		0.1 % phosphoric acid in water：methanol =90:10	280
Ellagic acid				1.0		0.1 % phosphoric acid in water：acetonitrile=80:20	254
Kaempferitrin				1.0		0.1 % phosphoric acid in water：acetonitrile=80:20	360

Table S2. HPLC LOD, LOQ, precision, stability and standard addition recovery test for the determination of 12 phenolic compounds.

metric name	Limit of detection (LOD, μg/mL)	Limit of quantification (LOQ, μg/mL)	Precision（RSD, %）	Stability(RSD, %)	Standard addition recovery test	
					Average recovery rate (%)	RSD (%)
Quercetin	0.0497	0.1657	0.13	0.71	96.85%	1.33%
Ethyl gallate	0.0357	0.1189	0.15	0.83	97.23%	1.59%
Gallic acid	0.0283	0.0944	0.14	0.75	97.85%	3.01%
Isoquercitrin	0.2062	0.6873	0.12	0.88	95.51%	1.65%
Quercitrin	0.3169	1.0563	0.18	0.69	94.47%	3.01%
Kaempferol	0.0593	0.1978	0.15	0.94	98.57%	0.94%
Rutin	0.0988	0.3292	0.11	0.85	95.91%	1.80%
Hyperoside	0.2293	0.7642	0.19	0.89	98.51%	1.79%
Tannic acid	0.4124	1.3746	0.12	0.74	96.47%	1.70%
Cianidanol	0.1138	0.3794	0.11	0.64	95.29%	2.57%
Ellagic acid	0.0190	0.0632	0.15	0.92	98.55%	1.51%
Kaempferitrin	0.3526	1.1753	0.15	0.71	95.70%	2.21%

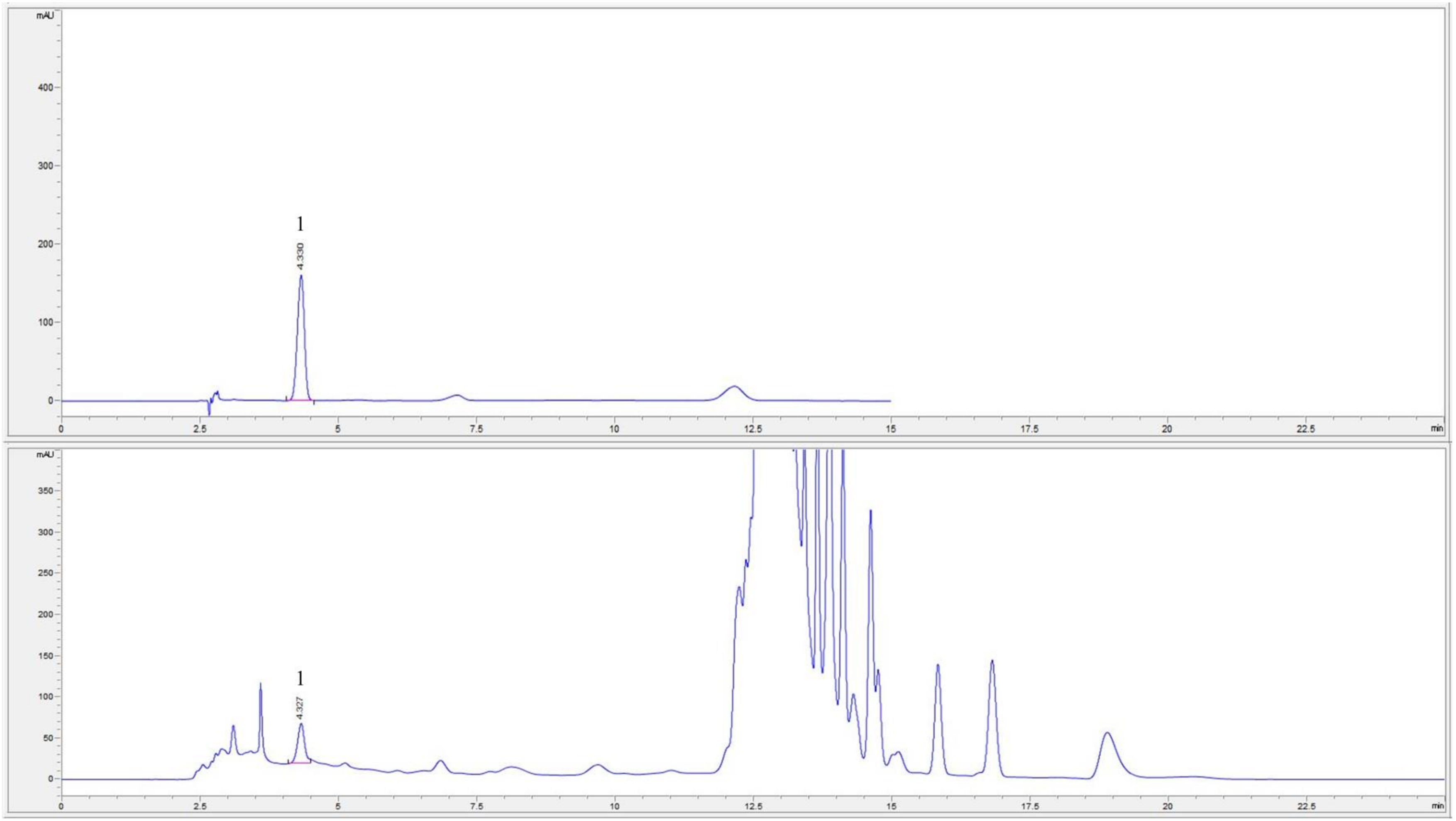

Figure S1 HPLC chromatogram of the authentic standards and *T. sebifera* leaves extract under optimum conditions. Peaks 1 is tannic acid.

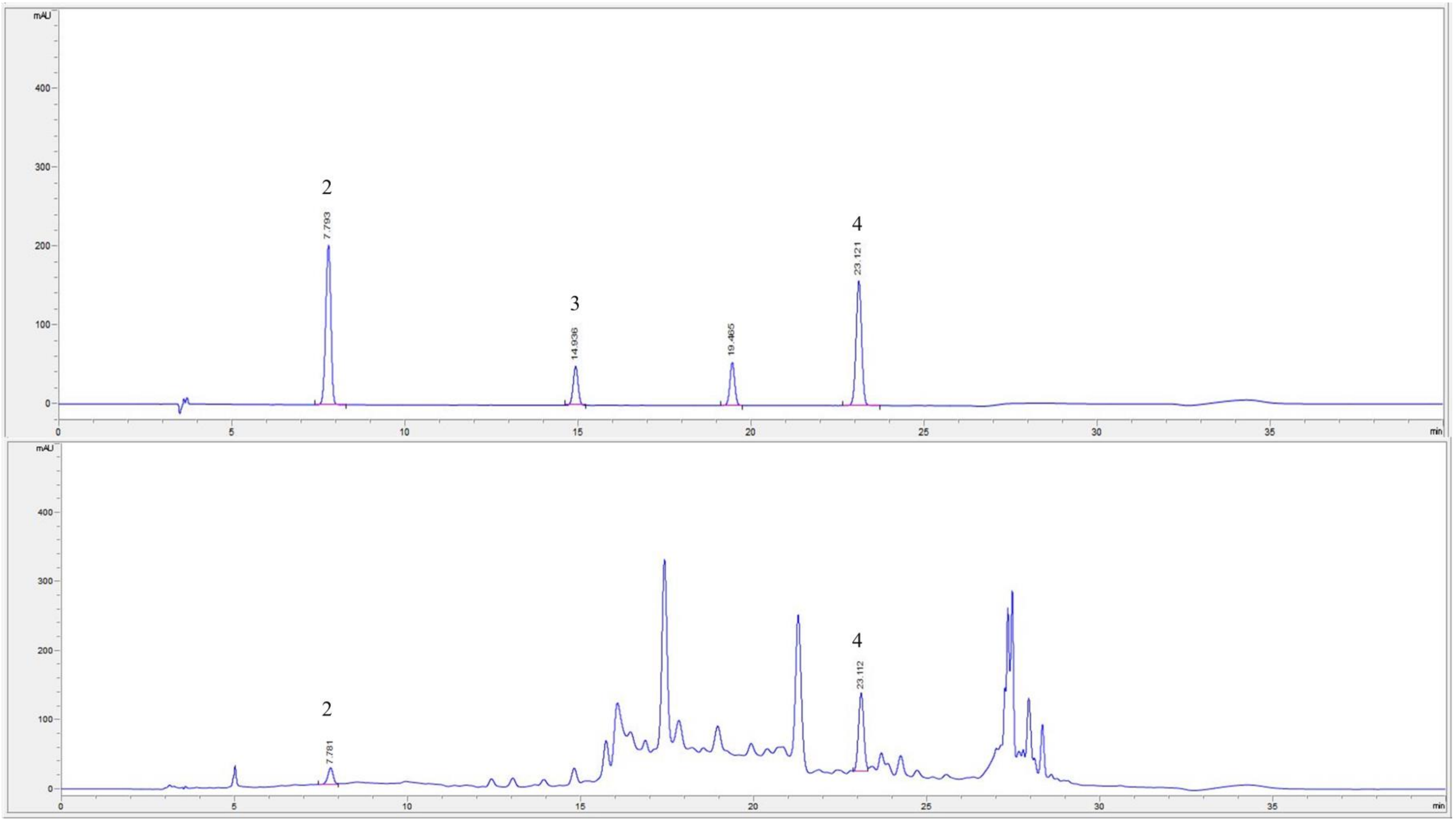

Figure S2 HPLC chromatogram of the authentic standards and *T. sebifera* leaves extract under optimum conditions. Peaks 2, 3 and 4 are gallic acid, cianidanol and ethyl gallate, respectively.

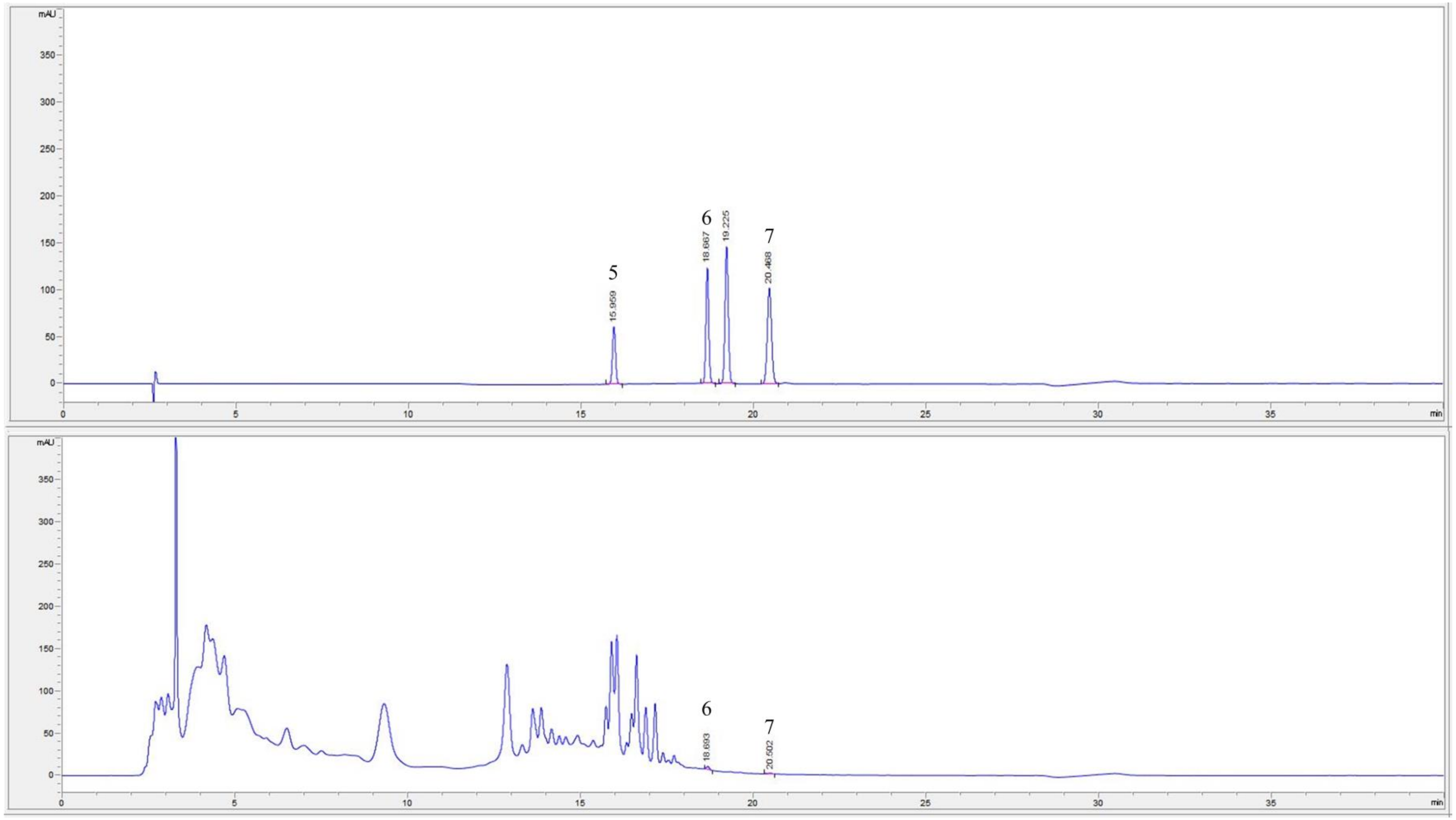

Figure S3 HPLC chromatogram of the authentic standardsand *T. sebifera* leaves extract under optimum conditions. Peaks 5, 6 and 7 are rutin, quercetinand kaempferol, respectively.

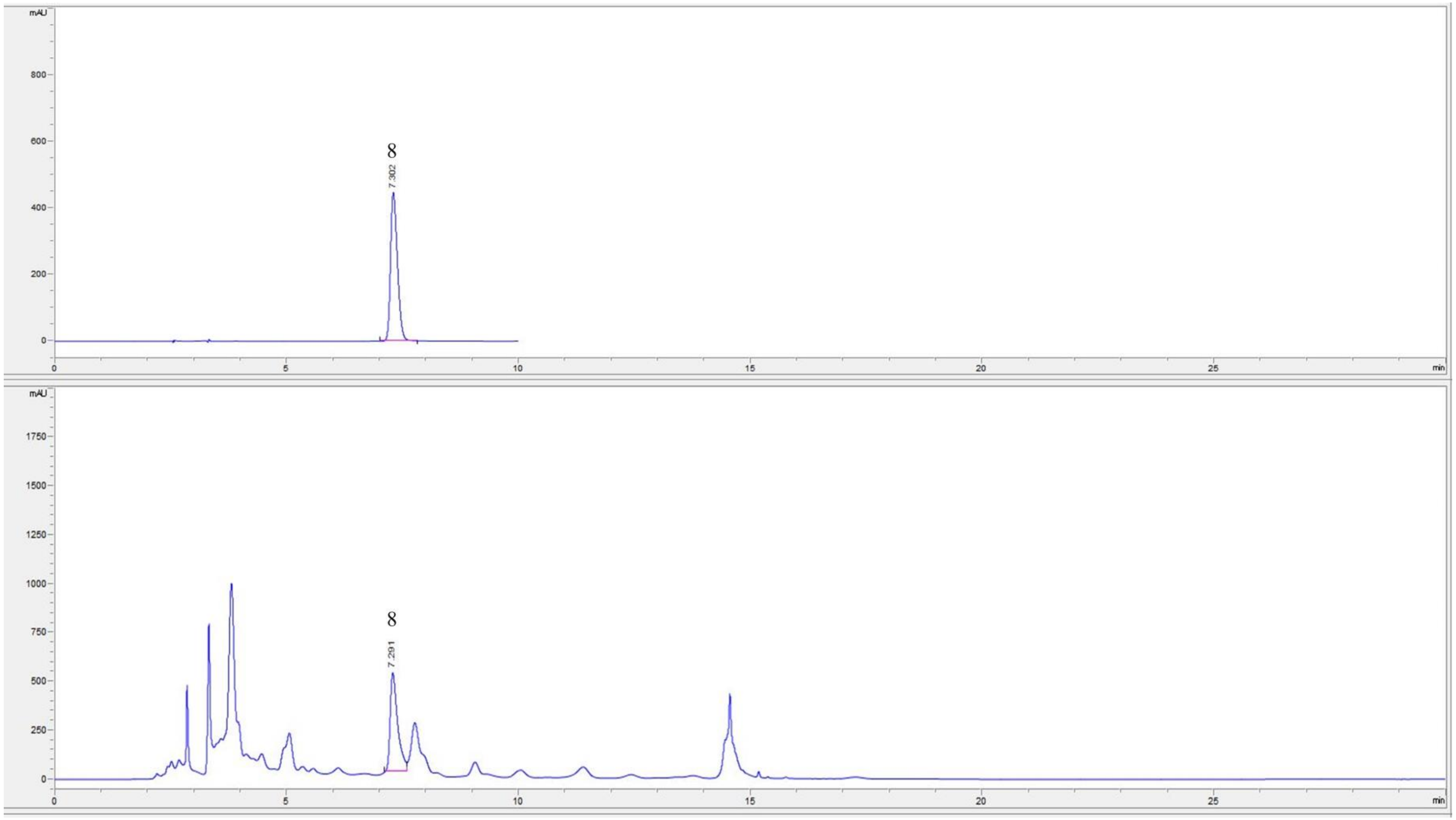

Figure S4 HPLC chromatogram of the authentic standards and *T. sebifera* leaves extract under optimum conditions.Peaks 8 is ellagic acid.

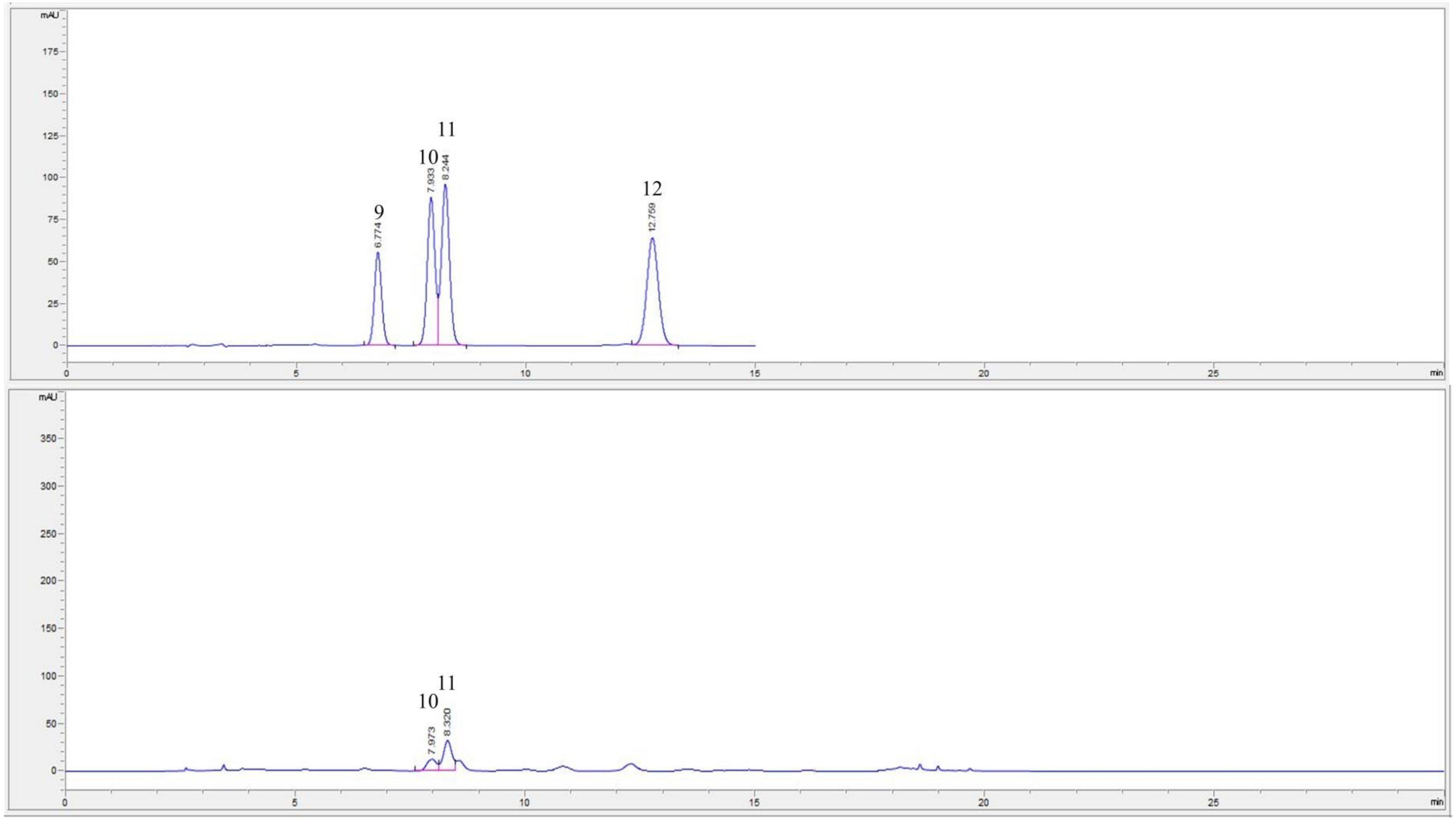

Figure S5 HPLC chromatogram of the authentic standards and *T. sebifera* leaves extract under optimum conditions. Peaks 9, 10, 11 and 12 are kaempferitrin, hyperoside, isoquercitrin and quercitrin, respectively.
